# The Latin American experience with a next generation sequencing genetic panel for recessive limb-girdle muscular weakness and Pompe disease

**DOI:** 10.1186/s13023-019-1291-2

**Published:** 2020-01-13

**Authors:** Jorge A. Bevilacqua, Maria del Rosario Guecaimburu Ehuletche, Abayuba Perna, Alberto Dubrovsky, Marcondes C. Franca, Steven Vargas, Madhuri Hegde, Kristl G. Claeys, Volker Straub, Nadia Daba, Roberta Faria, Magali Periquet, Susan Sparks, Nathan Thibault, Roberto Araujo

**Affiliations:** 1 Departamento de Neurología y Neurocirugía, Hospital Clínico, Universidad de Chile, Santiago, Chile; 2 Departamento de Anatomía y Medicina Legal, Facultad de Medicina, Universidad de Chile, Santiago, Chile; 3 Departamento de Neurología y Neurocirugía, Clínica Dávila, Santiago, Chile; 4 Genetics Department, UDELAR, Montevideo, Uruguay; 5 Institute of Neurology, Hospital de Clínicas, School of Medicine, UDELAR, Montevideo, Uruguay; 6 Institute of Neuroscience, Favaloro Foundation, Buenos Aires, Argentina; 7 Department of Neurology, University of Campinas-UNICAMP, Campinas, Sao Paulo Brazil; 8 Center of Neurology and Neurosurgery, Mexico City, Mexico; 9 Global Laboratory Services, Diagnostics, PerkinElmer, Waltham, MA USA; 10 Department of Neurology, University Hospitals Leuven, Leuven, Belgium; 11 Laboratory for Muscle Diseases and Neuropathies, Department of Neurosciences, KU Leuven, Campus Gasthuisberg, Leuven, Belgium; 12 John Walton Muscular Dystrophy Research Centre, Institute of Genetic Medicine, Newcastle University, Centre for Life, Newcastle, United Kingdom; 13 Sanofi, Dubai, United Arab Emirates; 14 Sanofi, Sao Paulo, Brazil; 15 Sanofi, Amsterdam, The Netherlands; 16 Sanofi Genzyme, Cambridge, MA USA

**Keywords:** Next-generation sequencing, Limb-girdle muscle weakness, Pompe disease, Latin America

## Abstract

**Background:**

Limb-girdle muscular dystrophy (LGMD) is a group of neuromuscular disorders of heterogeneous genetic etiology with more than 30 directly related genes. LGMD is characterized by progressive muscle weakness involving the shoulder and pelvic girdles. An important differential diagnosis among patients presenting with proximal muscle weakness (PMW) is late-onset Pompe disease (LOPD), a rare neuromuscular glycogen storage disorder, which often presents with early respiratory insufficiency in addition to PMW. Patients with PMW, with or without respiratory symptoms, were included in this study of Latin American patients to evaluate the profile of variants for the included genes related to LGMD recessive (R) and LOPD and the frequency of variants in each gene among this patient population.

**Results:**

Over 20 institutions across Latin America (Brazil, Argentina, Peru, Ecuador, Mexico, and Chile) enrolled 2103 individuals during 2016 and 2017. Nine autosomal recessive LGMDs and Pompe disease were investigated in a 10-gene panel (*ANO5*, *CAPN3*, *DYSF*, *FKRP*, *GAA*, *SGCA*, *SGCB*, *SGCD*, *SGCG*, *TCAP*) based on reported disease frequency in Latin America. Sequencing was performed with Illumina’s NextSeq500 and variants were classified according to ACMG guidelines; pathogenic and likely pathogenic were treated as one category (P) and variants of unknown significance (VUS) are described. Genetic variants were identified in 55.8% of patients, with 16% receiving a definitive molecular diagnosis; 39.8% had VUS. Nine patients were identified with Pompe disease.

**Conclusions:**

The results demonstrate the effectiveness of this targeted genetic panel and the importance of including Pompe disease in the differential diagnosis for patients presenting with PMW.

## Background

Limb-Girdle Muscular Dystrophy (LGMD) is a broad and heterogeneous category of inherited muscular diseases involving proximal muscle weakness in which the pelvic or scapular muscles are generally affected. The clinical evolution and phenotype vary widely and overlap, from severe forms with infantile onset and rapid progression to milder forms in which affected individuals have a slow progression and a relatively normal life [1].

LGMD is primarily divided into two major categories, based on inheritance pattern: LGMD D with autosomal dominant inheritance and LGMD R with autosomal recessive inheritance pattern. LGMD D encompasses 5 subtypes of LGMD (LGMD D1 to D5) while LGMD R comprises 24 recessive forms (LGMD R1 to R24), each of which is caused by pathogenic variants in different genes [2–4]. The autosomal dominant forms are rarer, accounting for less than 10% of muscular dystrophies, whereas autosomal recessive forms are much more frequent [1, 5]. The most common forms of LGMD R worldwide are the types LGMD R1 calpain3-related (MIM# 11420), LGMD R2 dysferlin-related (MIM# 603009), LGMD R5 γ-sarcoglycan-related (MIM# 608896), LGMD R3 α-sarcoglycan-related (MIM# 600119), LGMD R4 β-sarcoglycan-related (MIM# 600900), LGMD R6 δ-sarcoglycan-related (MIM# 601411), LGMD R9 FKRP-related (MIM# 606596), and LGMD R12 anoctamin5-related (MIM# 608662) [2, 5, 6]. These are estimated to affect 1:14,500 to 1:123,000 individuals worldwide [5–7]. There are currently no available treatments for LGMDs despite several ongoing clinical trials [6].

Pathological features of muscular dystrophies can be observed with a muscle biopsy, presenting as necrosis and regeneration of muscle fibers with various levels of fibrosis and infiltration of adipose tissue [2]. However, obtaining a definitive and timely diagnosis for some forms of LGMDs is challenging in spite of the genetic basis and Mendelian inheritance pattern [5]. This long diagnostic journey endured by LGMD patients is due to the variability in age of onset, severity, and disease progression as well as issues with genetic testing access worldwide [2, 5].

Although no longer classified as a muscular dystrophy of the autosomal recessive type 2 V (LGMD2V) [8] in the updated nomenclature for LGMD, Pompe disease (MIM# 232300), also known as Glycogen Storage Disease Type II, is a rare metabolic disease with a broad clinical spectrum and overlapping signs and symptoms to recessive LGMDs [9]. The estimated prevalence of Pompe disease varies from 1:40,000 to 1:60,000. Based on newborn screening, the prevalence may be even higher [10], depending upon ethnic and geographic factors. Pompe disease is caused by pathogenic variants in the *GAA* gene, which encodes acid α-glucosidase (GAA), an enzyme responsible for glycogen breakdown in the lysosome [11]. Glycogen accumulation in the lysosome can result in a clinical spectrum ranging from a rapidly progressive infantile-onset form of the disease (IOPD) to a more slowly progressive late-onset form referred to as late-onset Pompe disease (LOPD) [12]. In IOPD, the GAA activity is below 1% and infants present with severe cardiomyopathy, hypotonia, rapidly progressive muscle disease, and respiratory involvement. In LOPD, GAA activity is above 1% yet below 30% of average normal activity and symptom onset may occur at any age, usually without cardiomyopathy, but with progressive skeletal and respiratory muscle weakness [13–16]. The enzyme activity can be measured using fluorometry or mass spectrometry techniques in either lymphocyte or fibroblast cultures or as a screening test through dried blood spots (DBS) [17–19].

Since 2006, treatment with alglucosidase alfa (Myozyme®, Lumizyme®, Sanofi Genzyme, Cambridge, MA) has been approved for Pompe disease. Clinical trials have shown that treatment increases patient survival [20–26] and stabilizes respiratory and muscle function [26–30]. Early diagnosis is critical for the most effective treatment [16].

Genetic analysis for the identification of the altered gene is essential for the accurate and timely diagnosis of the LGMD R subtype as well as identification of patients with Pompe disease, which is part of the differential diagnosis in patients with proximal muscle weakness [2, 3]. The identification of variants in these Mendelian diseases, which is more straightforward due to inheritance patterns, can be a valuable component in the diagnosis of the disease and determining appropriate clinical and preventive procedures. Variants of unknown significance (VUS) may still present a challenge for diagnosis and may raise more questions in recessive disorders for patients with one or more VUS. Studies have shown that traditional techniques to identify protein abnormalities, such as immunohistochemistry, Western blotting, and Sanger sequencing for the identification of pathogenic variants, can yield a diagnosis of 35% of families with LGMD [3]. Western blotting and Sanger sequencing for Pompe disease have high specificity but low yield [31].

Targeted-panel next-generation sequencing (NGS) is leading to a paradigm shift in the diagnosis of many neuromuscular disorders, enabling individualized precision medicine. NGS allows the evaluation of several genes simultaneously, improving the diagnosis of Mendelian diseases that have a varied phenotype (eg, LGMD). NGS may increase the molecular diagnosis of LGMD R because it generates more data at a lower cost, accelerating the process of identification of pathogenic variants and new genes associated with Mendelian diseases [32, 33]. A growing number of studies using NGS have reported genes and variants associated with rare diseases [34–36]. These data are being compiled into databases of Mendelian diseases (OMIM) and variants with clinical significance (ClinVar) [37].

The prevalence of LGMD types varies in different geographical locations [5] and the success rate in diagnosis using NGS varies greatly between populations. To date, the success rate of sequencing of a gene panel for the diagnosis of LGMD R or LOPD has not been reported in the Latin American population. A recent study that looked at enzymatic activity showed a 4.2% yield for Pompe disease [9]; however, no study designed to assess variants in a Latin American population or how Pompe disease is related with other LGMD has been conducted. We investigated the sensitivity and specificity for the detection of variants in a gene panel associated with the most common forms of LGMD R and LOPD in a population with undiagnosed limb-girdle weakness in Latin America.

## Methods

### Sample

The study sample was a convenience sample from 20 institutions from Brazil, Mexico, Argentina, Chile, Peru, and Ecuador. Blood samples were from patients who underwent the genetic sequencing examination, with clinically suspected limb-girdle syndrome (proximal muscle weakness with or without respiratory symptoms) without confirmed diagnosis per molecular and/or immunohistochemical analysis. Serum creatine kinase activity was not part of the inclusion criteria. Included individuals had already received the results of the laboratory evaluation and were guided by their respective physicians, according to their clinical care practices. Individuals had not been tested for Pompe disease via a screening or enzymatic assay.

### Procedures

Peripheral DBS were collected on filter paper from patients in Latin America. The samples were received during 2016 and 2017 and without any information that allowed patient identification. The only identifying information available was the geographical origin of each sample. Samples were processed at DLE Laboratory, Sao Paulo, Brazil.

### Sequencing analysis

The NGS panel was chosen based on worldwide prevalence, national and regional epidemiology, and local technical capacity [1, 38, 39]. Variants were classified according to the criteria established by the American College of Medical Genetics and Genomics (ACMG) [40]. The ACMG established a scoring system using a series of criteria that are based on information about the variant (eg, protein effect, position in the transcript, literature information, functional assays, database, and prediction software). The presence or absence of certain traits is weighted differently, helping to determine whether the variant is pathogenic, probably pathogenic, or a variant of uncertain, probably benign, or benign significance. The chosen genetic panel with the coding regions and 10 nucleotides from the exon-intron junction from the included genes and intronic variants (Table 1) were customized with Agilent Sure-Select capture; this panel covers above 98% of target regions at 20x or greater. Nine genes and 154 corresponding exons related to muscular dystrophy and *GAA/*Pompe disease were included. Deep intronic variants were also targeted. Flanking exon/intron regions up to 25 base pairs (bp) were sequenced, as well as known intronic variants if outside of this range.

**Table 1:** Myopathies, transcripts, and deep intronic variants included in the NGS panel

Myopathy	Name	Gene	Transcript	Exons	Intronic Variants
LGMD R1 calpain3-related	Calpainopathy	*CAPN3*	NM_000070	24	1746-20C > G; 2264-11C > T; 2381-12A > G
LGMD R2 dysferlin-related	Dysferlinopathy	*DYSF*	NM_003494	55	-116delC; 2163-11G > A; 2355 + 14G > A; 3442 + 14C > T
LGMD R5 γ-sarcoglycan-related	γ-sarcoglycanopathy	*SGCG*	NM_000231	7	
LGMD R3 α-sarcoglycan-related	α-sarcoglycanopathy	*SGCA*	NM_000023	9	158-11G > A
LGMD R4 β-sarcoglycan-related	β-sarcoglycanopathy	*SGCB*	NM_000232	6	
LGMD R6 δ-sarcoglycan-related	δ-sarcoglycanopathy	*SGCD*	NM_000337	9	
LGMD R7 telethonin-related	Telethoninopathy	*TCAP*	NM_003673	2	
LGMD R9 FKRP-related		*FKRP*	NM_024301	1	
LGMD R12 anoctamin5-related		*ANO5*	NM_213599	22	
Pompe Disease (PD)		*GAA*	NM_000152	19	2481 + 16G > A; 2800-11C > G; -32-13 T > G
Total				154	

The coding and flanking intronic regions are enriched using a Custom SureSelect QXT kit (Agilent technology) and were sequenced using the Illumina NextSeq 500 system. The sequence reads were mapped to the human reference genome (hg19) using BWA software. Only variants (SNVs/Small Indels) in the coding region and the flanking intronic regions (+ 10 bp) with a minor allele frequency (MAF) < 5% are evaluated. The ExAC, 1000Genomes, and ABraOM projects were used to determine the frequency of the variants; CADD score over 20 was the threshold to classify the in silico damaging prediction of the variant to the final protein, and other published information and laboratory databanks were used to further classify the variants. Patients who had pathogenic variants in homozygous or compound heterozygous state for *GAA* consistent with Pompe disease had GAA activity measured in the same paper filter card by fluorometry.

### Data analysis

After sequencing, the base call generates “.bcl” files were converted to .fastq using the “bcl2fastq” script. The data were mapped against the reference sequence of the human genome (GRCh37 / hg19) with BWA software. The aligned file was then used for calling variants with the Samtools software, followed by annotation using the Variant Effect Predictor (VEP). “.Vcf” files annotated with VEP and in-house scripts were converted to tabulated tables and incorporated frequency information from variants already sequenced as well as Reactome and OMIM information.

### NGS quality analysis (data not shown)

Quality analysis of the sequencing and call of variants was done by “.fastq” and “.bam” files checked with Qualimap software. In addition, the average size of sequenced reads, aligned reads, transition rate, transversion, insertion, and deletion was surveyed. The nomenclature followed HGVS guidelines [41].

## Results

The demographics of the total sample of 2103 patients are described in Table 2. The sample was 53.7% male and the majority were 18 years of age or older (74%) with an age range of < 1 year to almost 97 years.

**Table 2:** Summary statistics for demographic characteristics and geographic regions^a^

Parameter	Statistics
Total N	2103
Male / Female, n (%)	1129 (53.7) / 974 (46.3)
Age (y), mean ± SD (min, max)	34.2 ± 19.7 (0.6. 96.6)
< 18 years of age, n (%)	547 (26)
≥ 18 years of age, n (%)	1556 (74)
Country, n (%)	
Brazil	1078 (51.3)
Mexico	690 (32.8)
Argentina	247 (11.7)
Chile	48 (2.3)
Peru	32 (1.5)
Ecuador	8 (0.4)

^a^ The patient sample was a convenience sample not a population-based sample. The number of patients from each country is not necessarily representative of the proportion of patients at risk

**Fig. 1:**
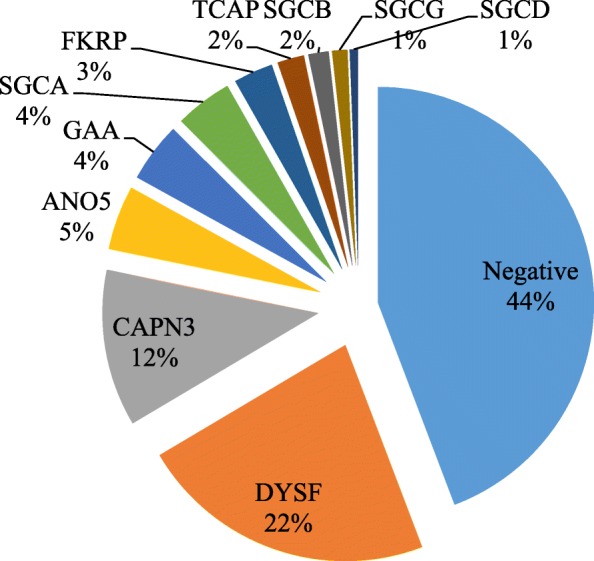
Percentages for each genetic variant and each intronic variant within the total population. 1173 (55.8%) patients had genetic variants identified by the panel

Of the 2103 patients, 1173 (55.8%) had genetic variants identified by the panel. Frequencies for each genetic variant and each intronic variant within the total population are described in Fig. 1. Targeted intronic variants represented 2.92% (45/1542) of all pathogenic variants and VUS. The largest proportion of these targeted intronic variants was found in *GAA* (30/45). No patient was homozygous for one of the included intronic variants.

**Fig. 2:**
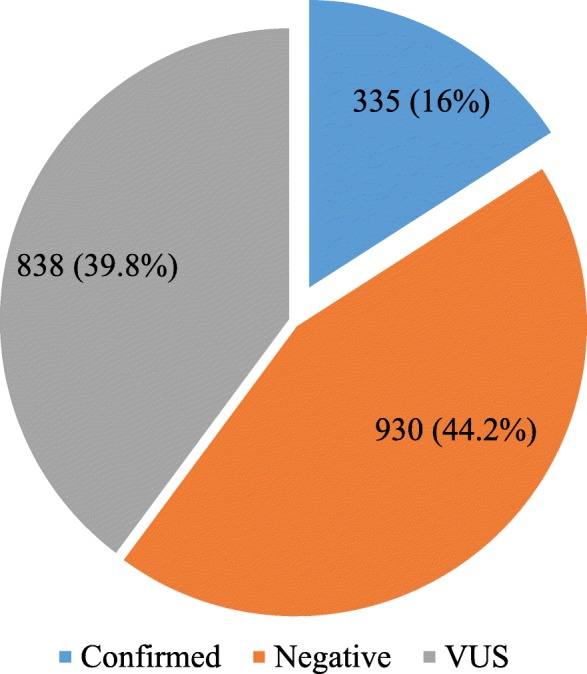
Frequencies and percentages of patients with confirmed molecular diagnosis, negative diagnosis, or variants of unknown significance (VUS)

In the total population, less than half of the samples were negative (*n* = 930, 44.2%), almost a third were identified with a VUS (*n* = 838, 29.8%), and 16% (*n* = 335) received a confirmed molecular diagnosis (homozygous or compound heterozygous) (Fig. 2). Table 3 shows the number of individuals with each disease out of the 335 with a confirmed molecular diagnosis. The majority were LGMD R2 (37.9%) and LGMD R1 (26.9%). Nine (2.7%) patients received a confirmed molecular diagnosis of Pompe disease, the eighth most frequent cause of LGMW in the cohort. The frequencies of variants among those who received a diagnosis are listed in Table 3, and the top 25 most frequent variants by gene in Latin America are listed in Table 4. In this list, variants in *GAA* were the third most frequent (24/335), after *DYSF* (39/335) and *SGCA* (29/335).

**Table 3:** Frequencies of variants among patients with any variant identified by the panel^a^

Gene	Variant Frequency, n (%)	Disease	Molecular Diagnosis Frequency, n (%) (*N* = 335)
*DYSF*	468 (39.89)	LGMD R2	127 (37.91)
*CAPN3*	247 (21.05)	LGMD R1	90 (26.87)
*ANO5*	101 (8.61)	LGMD R12	20 (5.97)
*GAA*	94 (8.01)	Pompe disease	9 (2.69)
*SGCA*	90 (7.68)	LGMD R3	33 (9.85)
*FKRP*	62 (5.29)	LGMD R9	18 (5.37)
*TCAP*	42 (3.58)	LGMD R7	15 (4.48)
*SGCB*	32 (2.73)	LGMD R4	10 (2.99)
*SGCG*	24 (2.05)	LGMD R5	9 (2.69)
*SGCD*	13 (1.12)	LGMD R6	4 (1.19)

^a^ Includes pathogenic variants and variants of uncertain significance (VUS)

**Table 4:** Most frequent pathogenic variants found by gene in Latin America (Top 25; N = 335 variants)

Rank	Gene	Status	DNA Variant	Protein
1	*DYSF*	Pathogenic	c.5979dupA (*n* = 38)	(p.Glu1994Argfs*3)
2	*SGCA*	Pathogenic	c.229C > T (*n* = 29)	(p.Arg77Cys)
3	*GAA*	Pathogenic	c.-32-13 T > G (*n* = 24)	p.?
4	*CAPN3*	Pathogenic	c.2362_2363delAGinsTCATCT (*n* = 18)	(p.Arg788Serfs*14)
5	*SGCA*	Pathogenic	c.850C > T (n = 18)	(p.Arg284Cys)
6	*TCAP*	Pathogenic	c.157C > T (n = 18)	(p.Gln53*)
7	*ANO5*	Pathogenic	c.172C > T (*n* = 15)	(p.Arg58Trp)
8	*SGCA*	VUS	c.421C > A (*n* = 13)	(p.Arg141Ser)
9	*DYSF*	Pathogenic	c.5429G > A (n = 13)	(p.Arg1810Lys)
10	*DYSF*	VUS	c.1402C > T (n-12)	(p.Arg468Cys)
11	*DYSF*	VUS	c.2281G > A (*n* = 11)	(p.Gly761Ser)
12	*CAPN3*	Pathogenic	c.328C > T (n = 11)	(p.Arg110*)
13	*SGCG*	Pathogenic	c.525del (n = 11)	(p.Phe175Leufs*20)
14	*DYSF*	Pathogenic	c.2777del (*n* = 11)	(p.Ala927Leufs*21)
15	*CAPN3*	VUS	c.2257G > A (*n* = 10)	(p.Asp753Asn)
16	*ANO5*	Pathogenic	c.692G > T (n = 8)	(p.Gly231Val)
17	*TCAP*	VUS	c.37_39del (n = 8)	(p.Glu13del)
18	*FKRP*	Pathogenic	c.826C > A (*n* = 7)	(p.Leu276Ile)
19	*CAPN3*	Pathogenic	c.1466G > A (n = 7)	(p.Arg489Gln)
20	*CAPN3*	Pathogenic	c.1468C > T (n = 6)	(p.Arg490Trp)
21	*GAA*	Pathogenic	c.2238G > C (n = 6)	(p.Trp746Cys)
22	*CAPN3*	Pathogenic	c.223dup (n = 6)	(p.Tyr75Leufs*5)
23	*ANO5*	Pathogenic	c.1359C > G (*n* = 5)	(p.Tyr453*)
24	*FKRP*	Pathogenic	c.1387A > G (n = 5)	(p.Asn463Asp)
25	*ANO5*	VUS	c.155A > G (n = 5)	(p.Asn52Ser)

Patients confirmed for Pompe disease (*n* = 9) had a mean age of 37 years (range: 15 to 56 years old), and 6 (66.7%) were female. The majority were heterozygous for the common IVS1 splice site variant, c.-32-13 T > G, in combination with known pathogenic variants. These patients with IVS1 splice variant had (1) a second deletion variant that results in a protein frameshift and termination at residue 45 of the GAA protein (c.525del [p.Glu176Argfs*45]) identified in a 42-year-old patient, (2) two nonsense mutations (c.2560C > T [p.Arg854*] mapping to exon 18, present in 2 siblings, and c.377G > A [p.Trp126*]) identified in patients 56, 64, and 42 years of age, (3) a missense mutation (c.1941C > G [p.Cys647Trp] mapping to exon 14) identified in a patient 28 years of age, and (4) a donor splice site variant resulting in a residue deletion (T > A transversion at the second nucleotide of intron 18 c.2646 + 2 T > A [p.Val876_Asn882del], also referred to as IVS18 + 2 T > A) identified in a patient 32 years of age. The youngest patient identified, 15 years of age, was heterozygous for a duplication that causes the insertion of a cysteine residue in exon 2 which results in a frameshift and premature stop codon (c.258dup [p.Asn87Glnfs*9]) and a missense variant (c.1445C > T [p.Pro482Leu]). Only 2 of the 9 patients diagnosed with Pompe disease carried homozygous variants, both missense type (c.1082C > T [p.Pro361Leu] mapping to the N-terminal B-sheet domain of the protein and c.1445C > T [p.Pro482Leu]), identified at 41 and 23 years of age, respectively.

The genotype IVS1 and c.2560C > T (p.Arg854*) was found in two sibling patients in this study. One patient was 54 years old with morning headaches and complaints of shortness of breath beginning at the age of 48. The second was a 56-year-old who presented with shortness of breath. Upon clinical investigation, the 54-year-old patient had a normal ECG, creatine kinase (CK) levels of 360IU/L, supine forced vital capacity of 28% and upright forced vital capacity of 47%, and a quadriceps biopsy with fiber size variability as the main finding and without signs suggestive of a glycogen storage disease. After a molecular diagnosis was made using the 10-gene panel, the enzymatic levels were tested and determined to be low for these patients.

The patients with no molecular diagnosis (44.2%) had (1) one heterozygous variant only, (2) two or more heterozygous variants in unrelated genes, or (3) one or two heterozygous and/or one homozygous VUS. Thirty-eight patients with one *GAA* variant identified by the panel were also screened by polymerase chain reaction for deletion of exon 18. One of the 38 patients negative for exon 18 deletion who was clinically suspected to have Pompe disease was also analyzed by multiplex ligation-dependent probe amplification and was found to be negative for large deletions elsewhere in *GAA*.

## Discussion

Over 8 years of data with over 1200 patients from approximately 220 families in North America, Europe, and Asia have demonstrated that NGS is an effective strategy for improving the diagnosis of patients with proximal muscle weakness [3, 5, 33, 36, 42–62] and identifying patients with Pompe disease among those with unclassified LGMD [31, 36, 63, 64]. The current study has now illustrated the effectiveness of NGS with the largest patient sample from a Latin American population. NGS identified genetic variants in 55.8% of 2103 tested patients and 16% of patients received a definitive molecular diagnosis. It is important to note that these results may not be representative of the of the regional incidence of the included forms of LGMD R and Pompe disease given that the study included only patients with proximal muscle weakness without a confirmed diagnosis and patients were not enrolled equally from each country.

Inclusion of *GAA* in the panel improved the overall performance in the identification of variants and in diagnostic yield. Four percent of the total population was identified with *GAA* variants, which were the fourth most frequently identified pathogenic variants (Table 4)*.* This compares favorably with the identification of other unclassified LGMD patients when *GAA* was included in the panel [17, 34, 35, 65]. Nine (2.7%) of the patients with a definitive molecular diagnosis were confirmed with Pompe disease.

Targeted deep intronic variants represented almost 3% of the total identified variants from this panel and were especially important in the identification of variants in the *GAA* gene and diagnosis of patients with Pompe disease. Among the 94 *GAA* variants, approximately one-third were intronic, and the majority of these intronic variants were the common IVS1 splice site variant. The inclusion of deep intronic variants allows for a more thorough genetic analysis and may help resolve cases that would otherwise remain unresolved in an exome-only NGS approach.

Our results are remarkably similar to other NGS programs reported in other geographic regions. The majority of variants identified in these other regional studies are similar and found within a limited set of genes in spite of diverse inclusion criteria and gene panels of varying size. In a study of 1001 European and Middle Eastern patients with undiagnosed limb-girdle muscle weakness and/or elevated serum CK activity, 20 genes of the 170-gene panel covered 80% of the patients for whom causal variants were found [66, 67]. Seven of the 10 genes included in the current study panel were among these top 20 genes—*CAPN3, DYSF, SGCG, SGCA, FKRP, ANO5,* and *GAA*. Eight patients from a European subset (*n* = 606) of these patients were identified with a *GAA* variant [67]. Similarly, in a large North American study of clinically suspected LGMD patients without molecular confirmation (*n* = 4656), 12 genes of the 35-gene NGS panel accounted for all of the patients with identified causal variants [6]. Eight of these genes were included in the 10-gene panel of the current study— *CAPN3, DYSF*, *FKRP*, *ANO5*, *SGCB, SGCA*, *GAA*, and *SGCB*. The molecular diagnostic yield for this study was 27%. The majority of patients with a molecular diagnosis had variants in *CAPN3* (17%), *DYSF* (16%), *FKRP* (9%), and *ANO5* (7%). Thirty-eight cases of LOPD were identified. Similar to our study, the vast majority (31/38) of the LOPD patients carried the IVS1 variant. The frequencies of gene variants in this Latin American population were similar to studies in other geographic regions, despite variability in inclusion criteria and size of the gene panel [17–19, 34, 36, 65, 68–70].

Across these geographically diverse, multigene panel testing studies, patients came from the United States, Canada, Europe, the Middle East, and now Latin America. The size of the gene panel for each study has varied from 10 in our study to 170 in the European/Middle Eastern study. The highest identification of variants (49%) was found with the largest panel [66, 67]. For the United States sample with the 35-gene panel, the identification of variants was 27% [6]. For the Canadian sample with a 98-gene panel, the identification of variants was 15%; however, the sample size for this study was only 34 patients [63]. Kuhn et al. evaluated 58 patients from Germany with clinical suspicion for LGMD and obtained a success rate of 33% using a 38-gene panel [33]. Similarly, a commercial panel containing the 9 genes associated with the most common forms of LGMD (LGMD R1, LGMD R2, rippling muscle disease, LGMD R3–6 and LGMD R9) had a diagnostic yield of 37% in a United States population [71]. Further studies are ongoing in Asia and the South Pacific. Two Asian populations have been evaluated. Dai et al. investigated 399 genes in patients with clinical diagnosis of muscular dystrophy and congenital myopathies and obtained a diagnostic yield of 65% of the patients [44]. Seong et al. evaluated a much smaller number of genes (18 genes) and obtained a similar diagnostic yield of 57% [57]. The current Latin American sample with a carefully selected 10-gene panel had a similar yield of identification of variants as the Canadian study (16%).

The diagnostic yield in the current study was lower than expected, possibly due to minimal entry criteria. The only inclusion criteria were limb-girdle weakness suggestive of LGMD and no molecular confirmation; elevated serum CK was not an inclusion criterion. A larger panel including more genes associated with diseases presenting with limb-girdle muscle weakness and/or more selective criteria for inclusion could improve the diagnostic yield, for example, the three “red flags” identified by Vissing et al. and also found by Preisler et al. in the three patients with proximal weakness diagnosed with Pompe disease in their study [65]. These three red flags are “1) mild non-dystrophic, myopathic features on muscle biopsy, often missing the typical vacuoles and glycogen accumulation, 2) CK levels below 1000, and 3) disproportionate axial and respiratory muscle involvement in comparison with limb muscle involvement.” Additionally, all reference databases have been developed with Caucasian populations and most of the populations studied have been European, North American, and Asian, which are known to be genetically more homogeneous than the Latin American population [3]. This may explain the large amount of VUS within this study. For these reasons, Latin American patients with 2 VUS and those with 1 pathogenic and 1 VUS should be investigated further.

The genotypes found for the newly identified LOPD patients are aligned with global experience, as the majority of these patients were heterozygous of the common splicing pathogenic variant IVS1. While clinical evaluation and follow-up data were limited for the patients diagnosed with Pompe disease in this study, these data were available for one of the two siblings with the genotype IVS1 and c.2560C > T. Despite inconclusive clinical findings, the 10-gene panel proved to be an effective differential diagnosis tool. Low GAA enzymatic activity levels further corroborated the diagnosis. Both patients with this genotype have not had access to treatment. The 54-year-old is being monitored continuously and has had slow disease progression in motor function and marked deterioration in respiratory function. Limited information is available for the older sibling. The disease progression of these patients is of interest because the disease is progressing differently for these siblings despite the same genotype and a similar environment [72–74].

There are several interesting observations concerning the genotypes and the age of the patients in which they were found. Three patients were below 30 years of age, including the 28-year-old with the IVS1 variant and the missense c.1941C > G. There is no reason to expect that the missense variant would lead to earlier signs and symptoms and more severe disease. However, no information is available on patient presentation. The youngest patient is a 15-year-old with the c.1445C > T and c.258dup genotype. Variant c.1445C > T maps to the catalytic GH31 domain of the GAA protein and was found in patients with symptom onset below 12 years of age and without cardiomyopathy in a global population [75]. Variant c.258dup was originally found in an IOPD patient from the United Kingdom and also identified in a 33-year-old North American patient by the 35-gene panel [6]. It is likely that the effect of the c.1445C > T mutation in combination with c.258dup may have led to early symptom presentation or increased disease severity, explaining the young age of the patients. We were also fortunate to identify a 23-year-old patient homozygous for c.1445C > T in this Latin America population.

The findings in this study demonstrate the importance of genetic testing for multiple diseases with overlapping phenotypes. In comparison to larger panels and panels with more defined inclusion criteria available in other regions, the 10-gene panel has performed reasonably well, albeit with somewhat lower yields. This could be due to several factors. One is the inherent limitation of the NGS technology applied. Other intronic variants, regulatory regions, modulatory genes and copy number variants are not considered. Thus, it is likely that a percentage of the unsolved cases are due to limitations in the technique applied. Other methods could be added to refine the investigation of unsolved cases. Secondly, given the high percentage of VUS variants across both Pompe disease and the 9 recessive LGMDs in the panel, further research into VUS variants found in this population is needed to possibly improve the diagnostic yield for Latin American patients. Thirdly, it is evident that increasing familiarity of the diagnostician with a simple limited panel such as the 10-gene panel is a positive way to support differential diagnosis, shorten patient journey to a definite diagnosis, and ultimately increase disease awareness.

## Conclusions

In this large cohort of Latin American patients, a simplified NGS strategy was effective for improving the diagnosis of patients with proximal muscle weakness. A genetic variant was identified in over half of the patients, with 16% receiving a definitive molecular diagnosis. The inclusion of *GAA* in the panel improved the overall diagnostic success, with 9 patients identified with Pompe disease (2.7% of patients with a confirmed diagnosis).

## Data Availability

Qualified researchers may request access to patient level data and related study documents including the clinical study report, study protocol with any amendments, blank case report form, statistical analysis plan, and dataset specifications. Patient level data will be anonymized, and study documents will be redacted to protect the privacy of trial participants. Further details on Sanofi’s data sharing criteria, eligible studies, and process for requesting access can be found at: https://www.clinicalstudydatarequest.com/.

## Abbreviations

ACMG: American College of Medical Genetics and Genomics
bp: Base pairs
CK: Creatine kinase
D: Dominant
DBS: Dried blood spot
GAA: Acid α-glucosidase
IOPD: Infantile-onset Pompe disease
LGMD: Limb-girdle muscular dystrophy
LOPD: Late-onset Pompe disease
MAF: Minor allele frequency
NGS: Next-generation sequencing
OMIM: Online Mendelian Inheritance in Man
P: Pathogenic
PD: Pompe disease
PMW: Proximal muscle weakness
R: Recessive
VEP: Variant effect predictor
VUS: Variants of unknown significance
